# Oromandibular Dystonia: Clinical and Demographic Data from Eight-Two Patients

**DOI:** 10.5334/tohm.730

**Published:** 2023-01-30

**Authors:** Mehmet Balal, Meltem Demirkiran

**Affiliations:** 1 Çukurova University School of Medicine, TR

**Keywords:** Oromandibular dystonia, clinical data, movement disorders

## Abstract

**Objective:**

This study aimed to determine the demographic and clinical characteristics of patients with oromandibular dystonia (OMD).

**Background:**

Dystonia is a movement disorder characterized by sustained involuntary muscle contractions that often cause abnormal postures. OMD is a rare focal dystonia that affects the tongue, jaw, and mouth. OMD, which is a rare public health problem, is often recognized as psychogenic and there are delays in its diagnosis and treatment.

**Methods:**

Patients with OMD, both isolated and combined, followed at our Movement Disorders Outpatient Clinic between 2004 and 2021 were enrolled in this study. Age, sex, age at onset, and disease duration were recorded. The type of OMD, affected muscles, etiologies of accompanying neurological disorders, and treatment were noted.

**Results:**

A total of 82 patients (44 women, 38 men) were included in this study. Among these, 39 patients had isolated OMD, and 43 patients had either segmental or generalized dystonia. Seven patients reported a family history of dystonia. Only nine patients reported a sensory trick. The average disease duration was 6.01 ± 3.73 (range, 1–29) years, and the average age at onset was 43.34 ± 18.24 (range, 1–78) years. The disease etiology was unknown (idiopathic) in most patients. Fifteen patients reported task-specific dystonia. The most common type of dystonia was jaw-opening dystonia.

**Conclusion:**

OMD is focal dystonia that significantly affects the quality of life. This study adds more data to the literature by defining the clinical features of this rare disorder and draws attention to this neglected type of dystonia.

## Introduction

Dystonia is a movement disorder characterized by abnormal and often recurrent involuntary continuous or intermittent muscle contractions. Dystonia is considered a network disorder that involves multiple brain regions including basal ganglia, cerebellum, thalamus, and other regions [1].

Dystonia is classified based on anatomical involvement or etiology [2]. Dystonia can be anatomically categorized according to the affected area as focal, segmental, multifocal, or generalized. Moreover, according to etiology, it can be classified as primary or secondary.

Oromandibular dystonia (OMD) is a rare focal dystonia that mainly affects the tongue, jaw, and mouth. OMD can be clinically divided into jaw opening (JO), jaw closing (JC), mixed (two or more combinations of JO, JC, or jaw deviation), lingual, and orobuccolingual subtypes. Although OMD is a focal dystonia, it may also be a part of segmental or generalized dystonia [2,3]. The annual incidence is reported to be between 3.3 and 6.9 per million people [4]. It often occurs after the fifth decade of life and is more common in women [4,5]. OMD is a chronic condition that affects speech, swallowing, and eating, causing deterioration in the quality of life [6]. Additionally, most patients experience pain. OMD is often recognized as psychogenic and there are delays in its diagnosis and treatment. This study aimed to determine the demographic and clinical characteristics of patients with OMD.

## Materials and Method

Patients with OMD monitored at our Movement Disorders Outpatient Clinic between 2004 and 2021 were included in this retrospective study. The faculty ethics committee approved this study.

Patients with a confirmed diagnosis of OMD who were regularly followed up at our clinic were included in the study. The patients included in the study were Turkish. Demographic data included age, sex, age at onset, and disease duration, whereas clinical data included OMD type, etiology, family history, affected muscles, sensory trick, task specificity, and accompanying movement disorders. The history, neurological examination, and brain imaging findings of all patients were reviewed to determine the etiology and type of dystonia. Also, the affected muscles were detected by two movement disorder specialists in double-blind form with electroneuromyography.

Statistical analysis was performed using IBM SPSS Statistics Version 24.0 (IBM Corp., Armonk, NY: IBM Corp.). Categorical measures were summarized as number and percentage and mean and standard deviation (median and minimum, where necessary) were used for numerical measurements. Numerical measurements were tested using the Shapiro–Wilk test, with the assumption of a normal distribution. The statistical significance was set at *p* < 0.05.

## Results

A total of 88 patients were identified. However, six patients whose diagnosis was not clear and who could not be followed-up regularly were excluded from the study. Finally, 82 patients (44 women and 38 men) were included in this study. The average age of the patients was 53.45 ± 16.73 (range, 18–89) years. The disease onset age was 43.34 ± 18.24 (range, 1–78) years and there was no significant difference in the age of onset for the different dystonia subtypes. The average disease duration was 10.11 ± 17.73 (range, 1–29) years. Demographic data are shown in Table 1.

**Table 1:** Demographic data of the patient.


	PATIENTS, N (%)	AGE, MEAN±SD (RANGE)	AGE AT ONSET, MEAN ± SD (RANGE)	DURATION OF DISEASE, MEAN ± SD (RANGE)

**Female**	44 (53.6)	55.8 ± 16.5 (19–89)	45.0 ± 19.3 (1–78)	10.7 ± 17.5 (1–29)

**Male**	38 (46.4)	50.5 ± 15.6 (18–78)	41.4 ± 16.6 (10–75)	9.1 ± 15.6 (1–20)

**Total**	82 (100)	53.4 ± 16.7 (18–89)	43.3 ± 18.2 (1–78)	10.1 ± 17.7 (1–29)

***p***	Nİ	Nİ	Nİ	Nİ


**Nİ**: No-significant, **SD**: Standart Deviation.

Seven patients (9%) had a family history of dystonia. These patients three had cervical dystonia and two had blepharospasm. The dystonia type of two patients could not be determined. Three and one patients had a family history of tremor and Parkinson’s disease, respectively. In the majority of patients (74.3%), the etiology was unknown (idiopathic). Idiopathic disease was more common in women than in men (79.6% vs 68.4%, p < 0.005).

The most common types were JO dystonia (47.5%) and lingual dystonia (42.6%), followed by JC, Orobuccolingal and mixed dystonia. Lingual dystonia was significantly associated with JO dystonia (43.8%). There were no significant differences between sexes. Isolated OMD, segmental dystonia, and generalized dystonia were observed in 39 (47.6%), 36 (43.9%) and 7 (8.5%) patients, respectively.

Blepharospasm was the most common segmental dystonia associated with OMD (14.6%). There was no correlation between the OMD subtypes and associated movement disorders. Depending on the type of OMD and associated movement disorders, many muscles were simultaneously affected. The most commonly affected muscles were the genioglossus (47.5%) and masseter (29.2%). There was no significant difference in the rate of involvement of other muscles. Task-specific dystonia was observed in 15 (18.2%) patients. Speech was the most commonly affected function (7.3%). Only nine (10.9%) patients reported a sensory trick. Patients aware of sensory trick were included in this group, no recommendations were made to the patients to develop sensory trick. The sensory tricks of the patients were chewing gum in 3, squeezing a napkin in the mouth in 3, using a toothpick in 2, and touching the chin in 2 of them. Clinical data are shown in Table 2.

**Table 2:** Clinical data of patients.


SEX	FEMALE (N, %)	MALE (N, %)	TOTAL (N, %)

**Patients**	44 (53.6)	38 (46.4)	82

**Family history**	7 (15.9)	3 (7.9)	10 (12.1)

OMD	4 (9.0)	3 (5.3)	7 (8.5)

Tremor	2 (4.5)	1 (2.6)	3 (3.6)

Parkinson’s Disease	1 (2.3)	–	1 (1.2)

**Etiology**	**n = 44**	**n = 38**	**n = 82**

Idiopathic	35 (79.6)	26 (68.4)	61 (74.3)

Tardive	5 (11.3)	3 (7.9)	8 (9.8)

Post-anoxic	2 (4.5)	4 (10.6)	6 (7.3)

Post-traumatic	1 (2.3)	3 (7.9)	4 (4.9)

Neurodegenerative	1 (2.3)	2 (5.2)	3 (3.7)

**OMD type**	**n = 44**	**n = 38**	**n = 82**

Jaw-opening	22 (50.0)	17 (44.7)	39 (47.5)

Jaw-closing	13 (29.5)	13 (34.2)	26 (31.8)

Mixed type	9 (20.5)	8 (21.1)	17 (20.7)

**Lingual**	21 (47.8)	14 (38.8)	35 (42.6)

**Oro-buccolingual**	12 (27.2)	6 (15.8)	18 (21.9)

**Associated movement disorders**	**n = 26**	**n = 17**	**n = 43**

Blepharospasm	7 (26.9)	5 (29.4)	12 (27.9)

Cranial dystonia	7 (26.9)	3 (17.7)	10 (23.3)

Facial dystonia	4 (15.4)	2 (11.7)	6 (13.9)

Cervical dystonia	3 (11.6)	3 (17.7)	6 (13.9)

Generalized dystonia	4 (15.4)	3 (17.7)	7 (16.3)

**Laryngeal dystonia**	1 (3.8)	1 (5.8)	2 (4.7)

**Affected muscles**	**n = 44**	**n = 38**	**n = 82**

Masseter	11 (25.0)	13 (34.2)	24 (29.2)

Genioglossus	24 (54.5)	15 (39.4)	39 (47.5)

Mentalis	6 (13.6)	5 (13.1)	11 (16.1)

Digastricus	7 (15.9)	5 (13.1)	12 (14.6)

Platysma	5 (11.3)	5 (13.1)	10 (12.1)

Nasalis	4 (9.0)	6 (15.7)	10 (12.1)

Orbicularis Oculi	7 (15.9)	5 (13.1)	12 (14.6)

Lateral Pterygoid	5 (11.3)	5 (13.1)	10 (12.1)

Temporalis	2 (4.5)	3 (7.8)	5 (6.0)

**Task-specific**	8 (18.2)	7 (18.4)	15 (18.2)

Speaking	3 (6.8)	3 (7.8)	6 (7.3)

Swallowing	3 (6.8)	2 (5.2)	5 (6.1)

Eating	2 (4.6)	2 (5.2)	4 (4.8)

**Sensory trick**	6 (13.6)	3 (7.8)	9 (10.9)


**OMD**: Oromandibular dystonia.

## Discussion

OMD is a rare disease, that decreases quality of life by affecting eating, drinking, speaking and swallowing functions [5]. In this study, the clinical and demographic data of 82 patients with OMD who were followed up at our outpatient clinic were determined. To the best of our knowledge, this is the largest cohort after those in the studies of Scorr et al., Slaim et al., Yoshida et al, Tan et al., [6,7,8,9]. In our study, OMD was more common in women, which is in accordance with the literature [2,3,6,7,8,9].

The onset of the disease is reported to be common after the 5th decade [6,7,8]. However, the age at onset was lower in our study. The lower mean age than the literature can be explained by the younger age of post-anoxic, post-traumatic and neurodegenerative patients. Moreover, men were younger than women. This may be because secondary etiologies were more common in men (31.6%) than in women (20.4%). However, this does not explain why OMD is more common among women. Although isolated OMD is rare, its association with other dystonias is relatively common. In our cohort, 47.6% of patients had focal OMD, 43.9% had segmental dystonia, and 8.5% presented with generalized dystonia. In the study by Slaim et al., the incidence of isolated OMD was 34.2% [6], which was lower than that in the present study. In our cohort, blepharospasm was the most common segmental dystonia associated with OMD (27.9%), which was similar to the findings of Slaim et al. (38.8%) [6]. Tan et al. found a greater association (50.0%) between blepharospasm and movement disorders [7]. Consistent with the literature, the most common type of OMD in this study was JO dystonia (47.5%) [6,7,8,9,10,11]. This was followed by JC and mixed dystonia (31.8% and 20.7%, respectively). Our data are consistent with those of Sinclair et al. (47.6%, 35.6%, and 16.9%) [8]. However, the JO dystonia rate was higher in other studies [6,7,11]. The prevalence of lingual dystonia was high among patients with OMD, particularly among patients with JO dystonia (43.8%). The data were consistent with those in the literature [6,7,8,9,10,11,12]. In addition, the association between JO and lingual dystonia caused a significant decrease in the quality of life of patients.

The most commonly affected muscle was the genioglossus (47.5%), consistent with the clinical findings of the patients. However, in most patients, more than one muscle was affected, complicating the findings. This made the diagnosis and treatment difficult. In the present study, 15 (18.2%) patients had task-specific dystonia. The most commonly affected function was speech (7.3%). Among patients with task-specific dystonia, two worked at a call center and one was a clergyman. These three patients were unable to work. Only nine (10.9%) patients reported a sensory trick.

## Conclusion

In our study, OMD was found to be more common in women and in the 5th decade. This study was conducted to add data to the existing sparse literature on the clinical features of this rare disease and draw attention to this neglected type of dystonia.

## Data Accessibility Statements

Data are available from the corresponding author upon reasonable request.

## References

[1] Albanese A, Bhatia K, Bressman SB, et al., “Phenomenology and classification of dystonia: a consensus update.” Mov Disord. 2013; 28: 863–873. DOI: 10.1002/mds.25475 23649720 PMC3729880

[2] Singer C, Papapetropoulos S. “A comparison of jaw-closing and jaw-opening idiopathic oromandibular dystonia.” Parkinsonism Relat Disord. 2006; 12: 115–118. DOI: 10.1016/j.parkreldis.2005.07.007 16271495

[3] Balasubramaniam R, Rasmussen J, Carlson LW, Van Sickels JE, Okeson JP. “Oromandibular dystonia revisited: a review and a unique case.” J Oral Maxillofac Surg. 2008; 66: 379–386. DOI: 10.1016/j.joms.2006.11.028 18201628

[4] Epidemiological Study of Dystonia in Europe (ESDE) Collaborative Group. “A prevalence study of primary dystonia in eight European countries.” J Neurol. 2000; 247: 787–792. DOI: 10.1007/s004150070094 11127535

[5] Merz RI, Deakin J, Hawthorne MR. “Oromandibular dystonia questionnaire (OMDQ-25): a valid and reliable instrument for measuring health-related quality of life.” Clin Otolaryngol. 2010; 35: 390–396. DOI: 10.1111/j.1749-4486.2010.02194.x 21108749

[6] Slaim L, Cohen M, Klap P, et al., “Oromandibular dystonia: demographics and clinical data from 240 patients.” J Mov Disord. 2018; 11(2): 78–81. DOI: 10.14802/jmd.17065 29860784 PMC5990905

[7] Tan EK, Jankovic J. “Botulinum toxin A in patients with oromandibular dystonia: long-term follow-up.” Neurology. 1999; 53: 2102–2107. DOI: 10.1212/WNL.53.9.2102 10599789

[8] Scorr LM, Factor SA, Parra SP, et al., ~Oromandibular Dystonia: A Clinical Examination of 2020 Cases.” Front Neurol. 2021; 12: 700714. DOI: 10.3389/fneur.2021.700714 34603182 PMC8481678

[9] Yoshida K. “Botulinum Neurotoxin Therapy for Lingual Dystonia Using an Individualized Injection Method Based on Clinical Features.” Toxins. 2019; 11(1): 51. DOI: 10.3390/toxins11010051 30658420 PMC6357149

[10] Sinclair CF, Gurey LE, Blitzer A. “Oromandibular dystonia: long-term management with botulinum toxin.” Laryngoscope. 2013; 123: 3078–3083. DOI: 10.1002/lary.23265 24122897

[11] Bakke M, Larsen BM, Dalager T, Moller E. “Oromandibular dystonia–functional and clinical characteristics: a report on 21 cases.” Oral Surg Oral Med Oral Pathol Oral Radiol. 2013; 115: 21–22. DOI: 10.1016/j.oooo.2012.04.023 22999966

[12] Papapetropoulos S, Singer CC. “Eating dysfunction associated with oromandibular dystonia: clinical characteristics and treatment considerations.” Head Face Med. 2006; 2: 47. DOI: 10.1186/1746-160X-2-47 17156419 PMC1702536

